# Preservation of Blood Flow to the Internal Iliac Artery Using a Custom-Made Single Fenestrated Endograft: A Case Report

**DOI:** 10.1177/15385744251315998

**Published:** 2025-02-11

**Authors:** Sara Shakery, Denise Nio, Maarten Truijers

**Affiliations:** 1 Department of Vascular Surgery, 3670 Spaarne Gasthuis, Haarlem, The Netherlands; 2 Department of Vascular Surgery, 26066 Amsterdam UMC, Amsterdam, The Netherlands

**Keywords:** internal iliac artery, custom-made device, fenestration, endograft, iliac branch, endovascular aneurysm repair, buttock claudication, ischemia, aneurysm

## Abstract

**Purpose:**

This report describes the use of a custom-made single-fenestrated endograft to preserve blood flow to the internal iliac artery (IIA) in a patient with an aorto-iliac aneurysm with unsuitable anatomy for a standard iliac branch device (IBD).

**Case Report:**

A 56-year-old man presented with an abdominal aortic aneurysm (AAA) of 56 mm involving the right common iliac artery (CIA). Use of a standard IBD for preservation of the IIA was deemed impossible due to narrow arrow anatomy of the right CIA. To preserve IIA flow, a custom-made Terumo endograft with an additional single-fenestration for the IIA was designed. The repair was successful and flow to the right IIA was preserved.

**Conclusion:**

Using a custom-made single-fenestrated endograft for the IIA in case of unsuitable anatomy for off-the-shelf IBDs prevents exclusion of the IIA and might prevent complications like buttock claudication and erectile dysfunction in patients with an aorto-iliac aneurysm. This report describes the use of a custom-made single fenestrated endograft to preserve blood flow to the IIA as a valuable alternative to standard iliac branched repair in patients with anatomical challenges.

## Introduction

A systematic review including 2671 patients reported that 15% of endovascular aneurysm repairs (EVARs) require internal iliac artery (IIA) occlusion which leads to buttock claudication in 27.9% of patients and erectile dysfunction in 10.2% of males.^1^ Patients experience less ischemic complications when 1 or both IIAs are preserved.^2^

To preserve IIA patency an iliac branch device (IBD) can be used in patients with suitable anatomy. In all off-the-shelf IBDs the diameter of the distal common iliac artery (CIA) needs to be at least 14 mm to prevent iliac limb compression. We describe the endovascular repair of an aorto-iliac aneurysm using a custom-made fenestrated Terumo (Terumo Aortic) endograft to preserve blood flow to the IIA.

## Case Report

**Figure 1.**
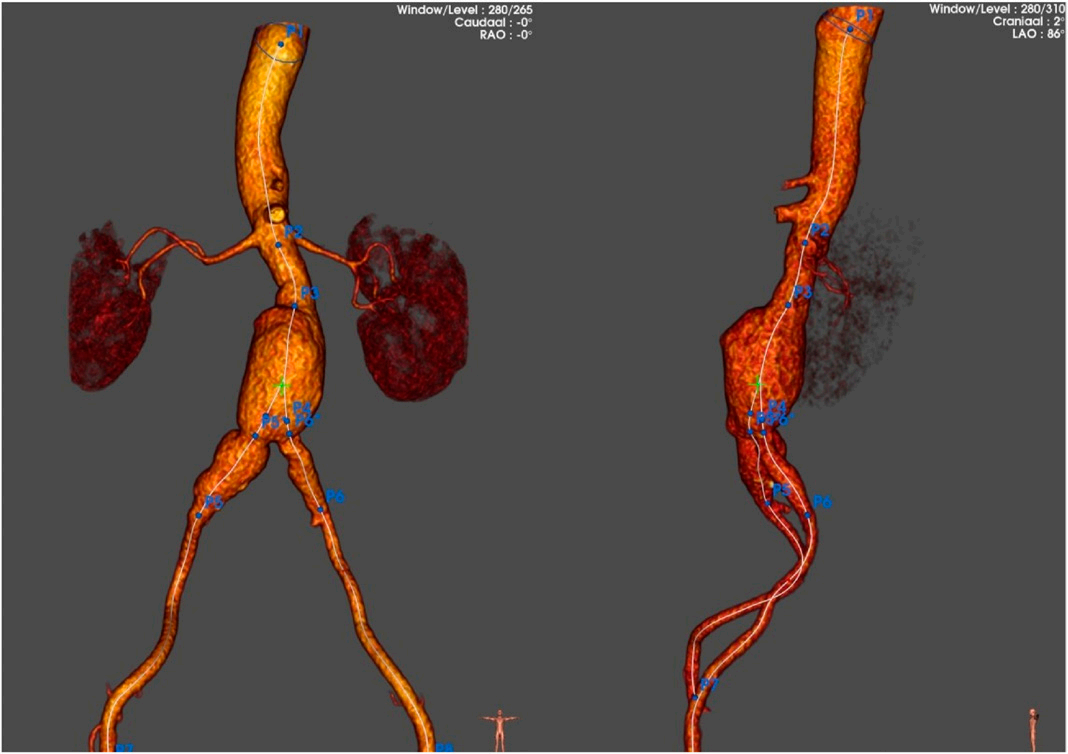
3D reconstruction of aorto-iliac aneurysm involving the right internal iliac artery.

A 56-year-old male with known coronary artery disease and previous popliteal aneurysm repair was under surveillance for an asymptomatic aortic aneurysm involving the right CIA (Figure 1). The patient did not exhibit symptoms of buttock claudication or erectile dysfunction during the surveillance period or before the intervention. During follow-up the abdominal aortic aneurysm (AAA) progressively increased in size to 56 mm. After reviewing the options, the patient preferred to undergo EVAR with maximum effort to preserve IIA flow.

**Figure 2.**
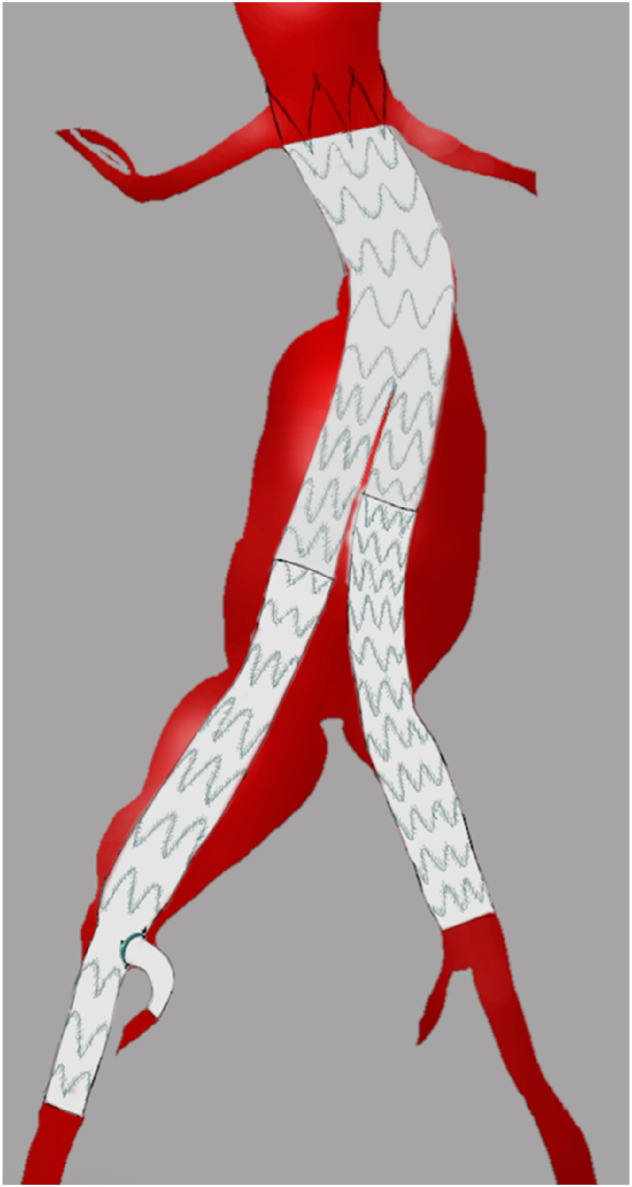
Reconstruction model of endograft with an additional single fenestration for the internal iliac artery.

**Figure 3.**
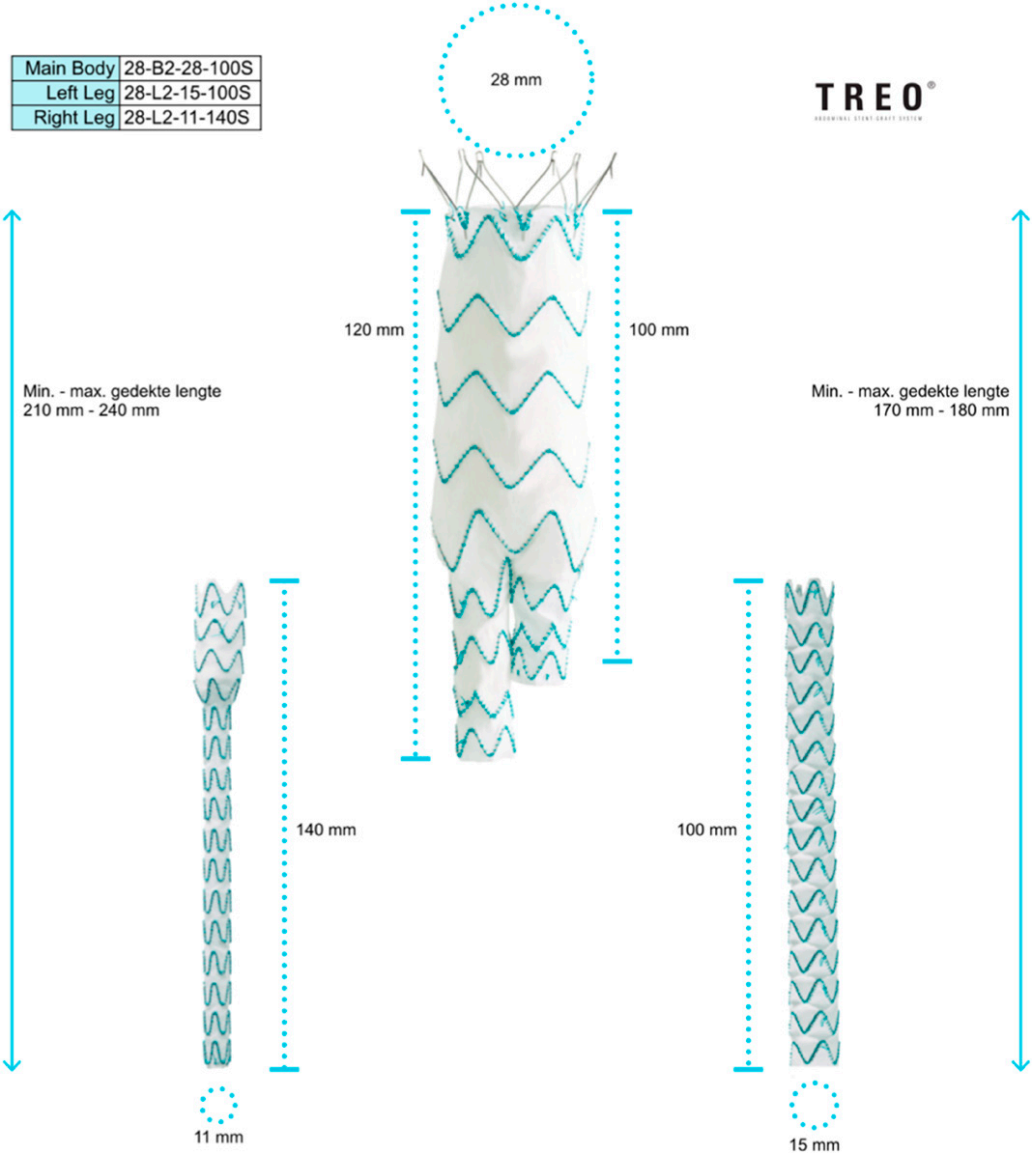
Reconstruction model of endograft designed by Terumo with corresponding sizes for main body, left and right leg.

The aortic aneurysm involved the right proximal and mid CIA. Standard endovascular iliac branched options to preserve IIA patency were deemed impossible because of a narrow distal CIA of 12 mm. To preserve IIA blood flow a custom-made Terumo endograft was designed. The endograft consisted of a standard tapered leg with an additional single-fenestration for the IIA (Figures 2 and 3).

The custom-made stent graft was handcrafted based on measurements of the specific anatomy of the patient. Technical drawings were created, detailing the specifications of the graft. The polyester graft was then tailored to fit a transparent template, incorporating nitinol stent rings. The fenestration for the IIA was made using cauterization and a nitinol fenestration ring was placed around the fenestration. The TREO endograft was selected because Terumo offers a wide range of custom-made options based on the regular EVAR platform. This allows for the deployment of the custom-made device in a reliable manner, minimizing potential deployment errors.

Percutaneous femoral access was established on each side. An off-the-shelf bifurcated Treo abdominal stent-graft (TREO 28-B2-28-100S) and iliac limb (TREO 28-L2-15-100S) were deployed. The ipsilateral limb was extended using the previously described custom-made fenestrated limb (TREO 15-L2-11-130 mm). After ipsilateral cannulation of the fenestration and IIA an Amplatz short tip guidewire (Boston Scientific) was used to introduce a 12 fr sheath into the IIA. Although a 7 French sheath could have been used, a 12 French sheath was chosen due to the stable positioning and the fact that the original graft was already placed through a larger sheath. A 6 mm × 59 mm × 80 cm Advanta V12 stent was advanced through the sheath and deployed after sheath withdrawal. The connection with the custom-made endograft was secured by flaring the V12 endograft using a plain PTA balloon (12 × 20 mm).

**Figure 4.**
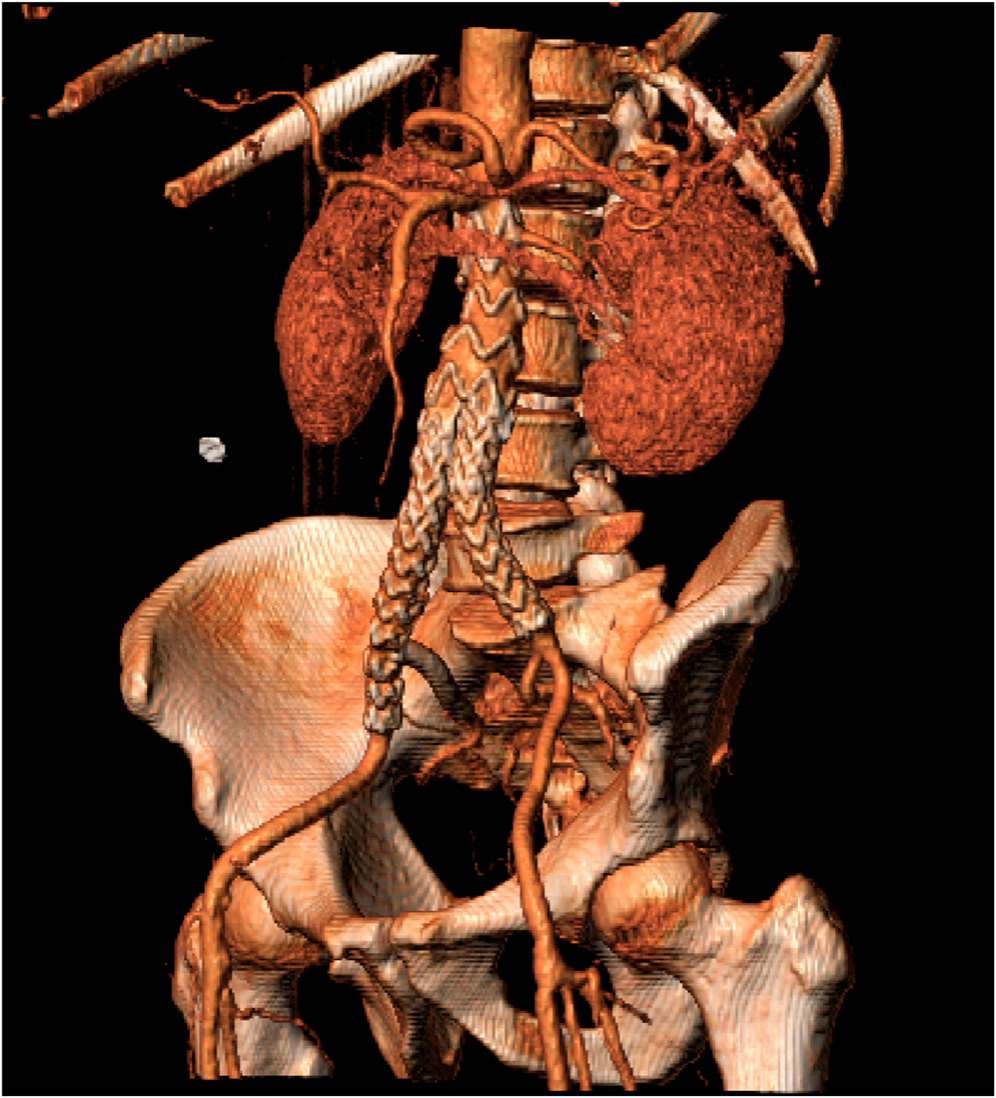
Follow-up computed tomography (CT) scan after 6 weeks (Figure shows reconstruction of the CT-scan. The raw CT-scan showed no signs of endoleak and the right internal iliac artery was patent).

Completion angiogram showed exclusion of the aorto-iliac aneurysm with preservation of blood flow into the IIA without endoleak. Follow-up computed tomography scan after 6 weeks and duplex ultrasound after 6 months demonstrated a successful repair with preserved flow to the IIA (Figure 4).

In the Netherlands, custom-made devices can be used in patients after obtaining their written informed consent. No additional Medical Ethics Review Committee approval is required. The patient provided written informed consent for the use of the custom-made stent graft.

## Discussion

Aorto-iliac aneurysms can be treated endovasculary while preserving at least 1 IIA using IBD, the sandwich technique, and surgeon-modified or in-situ laser fenestrations. Each of these techniques offers distinct advantages and limitations.

Standard off-the-shelf IBDs are used to preserve IIA patency in patients with suitable anatomy, requiring a CIA diameter of at least 14 mm to prevent stentgraft compression. A recent study by Cieri et al (2024) found that IBDs perform worse when the IIA diameter is ≥11 mm, with a higher incidence of type Ic endoleaks.^3^ Therefore, in cases where the CIA diameter is below 14 mm and/or the IIA diameter is 11 mm or smaller, such as the 12 mm distal CIA in the presented case, or in cases of anatomical abnormalities like tortuous or calcified iliac axes, a straight aortic bifurcation, or an aneurysmal or calcified IIA, alternative approaches are suggested.^4^

The sandwich technique is an alternative method for maintaining IIA patency, involving the placement of overlapping stents to create a channel for blood flow.^5^ While this technique can be effective, it carries risks such as potential for compression of 1 of the parallel grafts and potential for type III endoleak or stent migration.^6-8^

In advanced vascular centers, surgeon-modified fenestration and in-situ laser fenestration offer additional options for preserving IIA patency. These techniques involve creating fenestrations in situ by laser-generated fenestration, the sharp end of a guidewire, a needle or use of a radiofrequency probe to accommodate the iliac branch, providing flexibility in managing complex anatomies.^9-11^ Despite their potential benefits, these procedures require specialized skills and equipment, limiting their widespread adoption.

## Conclusion

EVAR using a custom-made fenestrated endograft offers a viable solution for preserving IIA patency in patients with aorto-iliac aneurysms and anatomy unsuitable for standard IBDs. This approach mitigates the limitations of off-the-shelf devices, such as the requirement for a minimum distal CIA diameter of 14 mm, and avoids the need for more complex techniques like the sandwich approach, surgeon-modified and in-situ laser fenestration.

This approach reduces the need to embolize or cover the IIA, potentially preventing ischemic complications such as buttock claudication and erectile dysfunction. As demonstrated in this case, custom-made devices can achieve successful aneurysm exclusion with preserved IIA flow, offering a valuable option for complex endovascular repairs.
